# Supplementary material for: The safety of combined triple drug therapy with ivermectin, diethylcarbamazine and albendazole in the neglected tropical diseases co-endemic setting of Fiji: A cluster randomised trial
Source: PLoS Negl Trop Dis. 2020 Mar 16;14(3):e0008106. doi: 10.1371/journal.pntd.0008106 (PMC7098623; doi:10.1371/journal.pntd.0008106)
Supplement: S2 Fig — AE: adverse event; PI: principal investigator; HREC: human research ethics committee. a Passive monitoring: participants asked to seek out study monitors if grade 1 symptoms worsen or new symptoms develop. All participants with symptoms higher than grade 1 will be actively followed. (PDF) [file pntd.0008106.s003.pdf]

S2 Fig. Adverse event monitoring flowchart

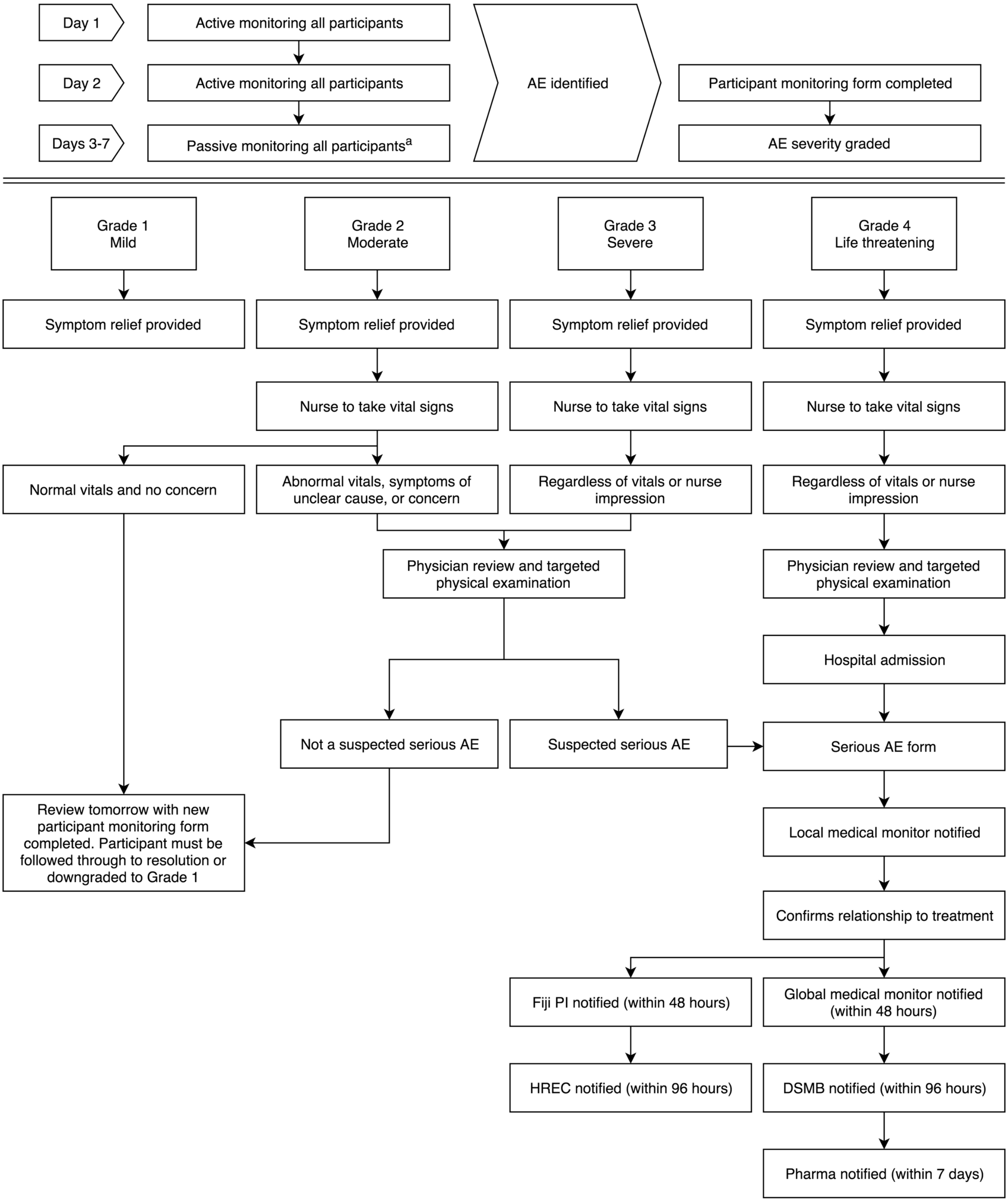

AE: adverse event; PI: principal investigator; HREC: human research ethics committee

[a] Passive monitoring: participants asked to seek out study monitors if grade 1 symptoms worsen or new symptoms develop. All participants with symptoms higher than grade 1 will be actively followed
