# Supplementary material for: The safety of combined triple drug therapy with ivermectin, diethylcarbamazine and albendazole in the neglected tropical diseases co-endemic setting of Fiji: A cluster randomised trial
Source: PLoS Negl Trop Dis. 2020 Mar 16;14(3):e0008106. doi: 10.1371/journal.pntd.0008106 (PMC7098623; doi:10.1371/journal.pntd.0008106)
Supplement: S1 Table — (PDF) [file pntd.0008106.s004.pdf]

S1 Table. Medication dosing schedule

MEDICATION DOSING CHART (by weight or age)	
**Albendazole**	
≥ 15 kg	1 tablet (400 mg)
**Diethylcarbamazine**	(dosing 6mg/kg)
15-25 kg	1 tablet (100 mg)
26-41 kg	2 tablets (200 mg)
42-58 kg	3 tablets (300 mg)
59-75 kg	4 tablets (400 mg)
76-92 kg	5 tablets (500 mg)
≥ 93 kg	6 tablets (600 mg)
**Ivermectin**	(dosing 200 μg/kg)
15-23 kg	1 tablet (3 mg)
24-38 kg	2 tablets (6 mg)
39-53 kg	3 tablets (9 mg)
54-68 kg	4 tablets (12 mg)
69-83 kg	5 tablets (15 mg)
84-98 kg	6 tablets (18 mg)
≥ 99 kg	7 tablets (21 mg)
**Permethrin**	(dosing topical 5%)
Age < 2 months	Apply for 4 hours
Age ≥ 2 months	Apply for 8 hours
