# Supplementary material for: The safety of combined triple drug therapy with ivermectin, diethylcarbamazine and albendazole in the neglected tropical diseases co-endemic setting of Fiji: A cluster randomised trial
Source: PLoS Negl Trop Dis. 2020 Mar 16;14(3):e0008106. doi: 10.1371/journal.pntd.0008106 (PMC7098623; doi:10.1371/journal.pntd.0008106)
Supplement: S2 Table — (PDF) [file pntd.0008106.s005.pdf]

S2 Table. Adverse event grading chart

Symptoms/Signs	Grades			
	1. Mild	2. Moderate	3. Severe	4. Life-threatening
Fever	38.0 – 39.0ºC	39.1 – 40.0ºC	> 40.0ºC	> 40.0ºC for > 48 hrs
Dizziness, giddiness, or fainting	Mild, not interfering with work or school	Moderate, unable to work or attend school for 1 day, but no fainting	Any loss of consciousness (fainting)	-
Headache	Mild pain not interfering with work or school	Moderate pain; pain or analgesics interfering with ability to work or attend school	Severe pain; pain or analgesics interfering with activities of daily living	Disabling, duration > 48 hrs.
Fatigue	Mild, not interfering with work or school	Moderate, unable to work or attend school at least 1 day	Severe, unable to perform activities of daily living, > 1day	Required hospitalization
Difficulty breathing (wheezing or dyspnea)	Mild, not interfering with work or school	Moderate, unable to work or attend school for 1 day	Severe, more than 1 day and required transfer to clinic or hospital	Hospitalization or respiratory failure requiring mechanical ventilation
Cough	Mild, relieved by non-prescription medication	Requiring narcotic antitussive	Severe cough or coughing spasms, poorly controlled by treatment	Hospitalization or respiratory failure requiring mechanical ventilation
Joint or muscle pain	Mild pain not interfering with work or school	Moderate pain; pain or analgesics interfering with ability to work or attend school	Severe pain; pain or analgesics interfering with activities of daily living	Disabling, duration > 48 hrs.
Muscle Weakness	Mild, not interfering with work or school	Moderate, unable to work or attend school at least 1 day	Severe, unable to perform activities of daily living, > 1day	Required hospitalization
Rash	Localized rash (covers only one part of the body)	Diffuse rash (covers multiple parts of the body)	Diffuse rash (covers multiple parts of the body) AND has any blisters or ulcers or mouth sores	Extensive areas with blisters or ulcers OR peeling or blackening of skin
Itching skin	Mild, not interfering with work or school	Moderate, unable to work or attend school 1 day	Severe, unable to work/school >1 day	Disabling >48hrs
Pain of skin	Mild, not interfering with work or school	Moderate, unable to work or attend school 1 day	Severe, unable to work/school >1 day	Disabling >48hrs
Swollen or painful nodes (armpit or groin)	Mild, not interfering with work or school	Moderate, unable to work or attend school 1 day	Severe, unable to work/school >1 day	Severe, limiting activities of daily living (unable to walk) > 2 days
Men only: testicular or scrotal pain	Mild, not interfering with work or school	Moderate, unable to work or attend school 1 day	Severe, unable to work/school >1 day	Severe, limiting activities of daily living (unable to walk) > 2 days
Nausea	Able to eat	Oral intake significantly decreased	No significant intake, requiring IV fluids	-
Vomiting	1 episode in the last 24 hours	2-5 episodes in the last 24 hours	≥ 6 episodes in the last 24hours, or need for IV fluids (Outpatient)	Hemodynamic collapse or overnight hospitalization
Diarrhea	Increase of < 4 stools/day over pre-treatment	Increase of 4-6 stools/ day, or nocturnal stools	Increase of ≥ 7 stools/ day or need for outpatient parenteral support for dehydration	Physiologic consequences with hemodynamic collapse or requiring hospitalization

Symptoms/Signs	Grades			
	**1. Mild**	**2. Moderate**	**3. Severe**	**4. Life-threatening**
Abdominal pain	Mild pain not interfering with work or school	Moderate pain; pain or analgesics interfering with ability to work or attend school	Severe pain; pain or analgesics interfering with activities of daily living	Disabling, duration > 48 hrs.
Unusual swelling (beyond baseline lymphedema)	Mild, not interfering with work or school	Moderate, unable to work or attend school 1 day	Severe, unable to work/school >1 day	Severe, limiting activities of daily living (unable to walk) > 2 days
Other illness or symptoms	Mild, not interfering with work or school	Moderate, unable to work or attend school at least 1 day	Unable to perform activities of daily living, > 1day	Required hospitalization
