# Supplementary material for: The safety of combined triple drug therapy with ivermectin, diethylcarbamazine and albendazole in the neglected tropical diseases co-endemic setting of Fiji: A cluster randomised trial
Source: PLoS Negl Trop Dis. 2020 Mar 16;14(3):e0008106. doi: 10.1371/journal.pntd.0008106 (PMC7098623; doi:10.1371/journal.pntd.0008106)
Supplement: S3 Table — (PDF) [file pntd.0008106.s006.pdf]

S3 Table. CONSORT 2010 checklist of information to include when reporting a cluster randomised trial

Section/Topic	Item No	Standard Checklist item	Extension for cluster designs	Section, Paragraph
**Title and abstract**				
	1a	Identification as a randomised trial in the title	Identification as a cluster randomised trial in the title	Title
	1b	Structured summary of trial design, methods, results, and conclusions (for specific guidance see CONSORT for abstracts)	See additional abstract table below	Abstract
**Introduction**				
**Background and objectives**	2a	Scientific background and explanation of rationale	Rationale for using a cluster design	Introduction
	2b	Specific objectives or hypotheses	Whether objectives pertain to the cluster level, the individual participant level or both	Introduction, Para 5
**Methods**				
**Trial design**	3a	Description of trial design (such as parallel, factorial) including allocation ratio	Definition of cluster and description of how the design features apply to the clusters	Methods, Para 4
	3b	Important changes to methods after trial commencement (such as eligibility criteria), with reasons		N/A
**Participants**	4a	Eligibility criteria for participants	Eligibility criteria for clusters	Methods, Para 4<br>Methods, Para 8
	4b	Settings and locations where the data were collected		Methods, Para 2 & 3
**Interventions**	5	The interventions for each group with sufficient details to allow replication, including how	Whether interventions pertain to the cluster level, the individual participant level or both	Methods, Para 1<br>Methods, Para 5-7

		and when they were actually administered		
**Outcomes**	6a	Completely defined pre-specified primary and secondary outcome measures, including how and when they were assessed	Whether outcome measures pertain to the cluster level, the individual participant level or both	Methods, Para 9-11<br><br>Methods, Para 13-15
	6b	Any changes to trial outcomes after the trial commenced, with reasons		N/A
**Sample size**	7a	How sample size was determined	Method of calculation, number of clusters(s) (and whether equal or unequal cluster sizes are assumed), cluster size, a coefficient of intracluster correlation (ICC or *k*), and an indication of its uncertainty	Methods, Para 4<br><br>Methods, Para 18
	7b	When applicable, explanation of any interim analyses and stopping guidelines		N/A
**Randomisation:**				
**Sequence generation**	8a	Method used to generate the random allocation sequence		Methods, Para 4
	8b	Type of randomisation; details of any restriction (such as blocking and block size)	Details of stratification or matching if used	Methods, Para 4
**Allocation concealment mechanism**	9	Mechanism used to implement the random allocation sequence (such as sequentially numbered containers), describing any steps taken to conceal the sequence until interventions were assigned	Specification that allocation was based on clusters rather than individuals and whether allocation concealment (if any) was at the cluster level, the individual participant level or both	Methods, Para 4
**Implementation**	10	Who generated the random allocation sequence, who enrolled participants, and who	Replace by 10a, 10b and 10c	

		assigned participants to interventions		
	10a		Who generated the random allocation sequence, who enrolled clusters, and who assigned clusters to interventions	Methods, Para 4
	10b		Mechanism by which individual participants were included in clusters for the purposes of the trial (such as complete enumeration, random sampling)	Methods, Para 4<br><br>Methods, Para 8
	10c		From whom consent was sought (representatives of the cluster, or individual cluster members, or both), and whether consent was sought before or after randomisation	Methods, Para 4<br><br>Methods, Para 8
**Blinding**	11a	If done, who was blinded after assignment to interventions (for example, participants, care providers, those assessing outcomes) and how		N/A
	11b	If relevant, description of the similarity of interventions		N/A
**Statistical methods**	12a	Statistical methods used to compare groups for primary and secondary outcomes	How clustering was taken into account	Methods, Para 17
	12b	Methods for additional analyses, such as subgroup analyses and adjusted analyses		Methods, Para 17
**Results**				
**Participant flow (a diagram is strongly recommended)**	13a	For each group, the numbers of participants who were randomly assigned, received intended treatment, and	For each group, the numbers of clusters that were randomly assigned, received intended treatment, and	Results, Para 1<br><br>Fig 1, Table 1, S4 Table<br><br>Results, Para 5 & 6

		were analysed for the primary outcome	were analysed for the primary outcome	
	13b	For each group, losses and exclusions after randomisation, together with reasons	For each group, losses and exclusions for both clusters and individual cluster members	Results, Para 1<br><br>Fig 1, S5 Table<br><br>Results, Para 5 & 6
**Recruitment**	14a	Dates defining the periods of recruitment and follow-up		Results, Para 1
	14b	Why the trial ended or was stopped		N/A
**Baseline data**	15	A table showing baseline demographic and clinical characteristics for each group	Baseline characteristics for the individual and cluster levels as applicable for each group	Table 1,<br><br>S4 Table, S6 Table
**Numbers analysed**	16	For each group, number of participants (denominator) included in each analysis and whether the analysis was by original assigned groups	For each group, number of clusters included in each analysis	Fig 1, Table 1<br><br>S4 Table, S6 Table<br><br>Results, Para 1 & 5
**Outcomes and estimation**	17a	For each primary and secondary outcome, results for each group, and the estimated effect size and its precision (such as 95% confidence interval)	Results at the individual or cluster level as applicable and a coefficient of intracluster correlation (ICC or k) for each primary outcome	Results, Para 2 & 3<br><br>Results, Para 6-7<br><br>Results, Para 12<br><br>Table 1 & 2, Fig 2<br><br>S6 Table, S7 Table
	17b	For binary outcomes, presentation of both absolute and relative effect sizes is recommended		N/A
**Ancillary analyses**	18	Results of any other analyses performed, including subgroup analyses and adjusted analyses, distinguishing pre-specified from exploratory		Results, Para 8-11<br><br>Results, Para 13<br><br>Table 3
**Harms**	19	All important harms or unintended effects in each group (for specific		Results, Para 6 & 7

		guidance see CONSORT for harms[1])		Table 2, Fig 2<br>Results, Para 12<br>S6 Table, S7 Table
**Discussion**				
**Limitations**	20	Trial limitations, addressing sources of potential bias, imprecision, and, if relevant, multiplicity of analyses		Discussion, Para 3<br>Discussion, Para 5
**Generalisability**	21	Generalisability (external validity, applicability) of the trial findings	Generalisability to clusters and/or individual participants (as relevant)	Discussion, Para 6 & 7
**Interpretation**	22	Interpretation consistent with results, balancing benefits and harms, and considering other relevant evidence		Discussion, Para 1-3<br>Discussion, Para 6 & 7
**Other information**				
**Registration**	23	Registration number and name of trial registry		Methods, Para 19
**Protocol**	24	Where the full trial protocol can be accessed, if available		S1 Protocol
**Funding**	25	Sources of funding and other support (such as supply of drugs), role of funders		Abstract, Para 5 & 6
