# Supplementary material for: The safety of combined triple drug therapy with ivermectin, diethylcarbamazine and albendazole in the neglected tropical diseases co-endemic setting of Fiji: A cluster randomised trial
Source: PLoS Negl Trop Dis. 2020 Mar 16;14(3):e0008106. doi: 10.1371/journal.pntd.0008106 (PMC7098623; doi:10.1371/journal.pntd.0008106)
Supplement: S4 Table — DA: diethylcarbamazine and albendazole; IDA1: ivermectin one dose, diethylcarbamazine and albendazole; IDA2: ivermectin two dose with DA; IQR: interquartile range. (PDF) [file pntd.0008106.s007.pdf]

S4 Table. Population and participant demographics by village

	Population Demographics					Participant Demographics					
	Total	Male		Age		Total		Male		Age	
Village	N	n	%	Median	IQR	N	%	n	%	Median	IQR
DA											
2	18	9	50.0	52.5	15-65	18	100	9	50.0	52.5	15-65
3	204	106	52.0	31	11-48.5	167	81.9	81	48.5	26	10-48
4	107	54	50.5	35	10-53	84	78.5	43	51.2	31.5	9-48
8	102	51	50.0	43.5	14-60	78	76.5	38	48.7	42.5	13-57
16	98	46	46.9	29	11-51	86	87.8	41	47.7	27	10-49
18	253	132	52.2	25	9-38	215	85.0	111	51.6	22	9-37
26	297	159	53.5	23	11-48	266	89.6	135	50.8	22	10-48
29	298	160	53.7	22	9-42	169	56.7	96	56.8	21	9-41
30	75	35	46.7	36	24-54	67	89.3	32	47.8	36	23-55
32	118	71	60.2	31.5	10-53	106	89.8	64	60.4	27.5	10-50
34	46	29	63.0	32	21-53	37	80.4	23	62.2	36	21-55
Total DA	1616	852	52.7	28.5	11-49	1293	80.0	673	52.0	27	10-48
IDA1											
6	79	40	50.6	38	12-55	67	84.8	35	52.2	33	10-53
7	66	37	56.1	31.5	13-44	47	71.2	27	57.4	23	11-40
10	240	129	53.8	31	12.5-52	204	85.0	109	53.4	29	12-50
13	73	31	42.5	44	16-69	59	80.8	25	42.4	36	13-55
15	151	73	48.3	31	13-52	126	83.4	64	50.8	24	12-46
17	86	49	57.0	30	12-51	78	90.7	46	59.0	23.5	12-49
19	99	59	59.6	25	10-44	84	84.8	47	56.0	22.5	9-42.5
22	139	66	47.5	31	8-52	116	83.5	49	42.2	31	8-51
24	45	26	57.8	38	29-53	40	88.9	23	57.5	42.5	29.5-59
25	92	41	44.6	31	8-51	75	81.5	34	45.3	20	8-50
27	146	74	50.7	26	9-45	127	87.0	62	48.8	23	9-44
35	160	78	48.8	16	15-18	159	99.4	78	49.1	16	15-18
Total IDA1	1376	703	51.1	26	12-49	1182	85.9	599	50.7	22	11-46
IDA2											
1	107	57	53.3	24	10-37	88	82.2	47	53.4	16	9-36
5	224	120	53.6	32	11-52	180	80.4	97	53.9	27	11-48.5
9	60	31	51.7	33	10-52.5	48	80.0	25	52.1	20	9-47
11	197	94	47.7	32	12-52	141	71.6	63	44.7	26	10-47
12	142	70	49.3	34.5	17-60	114	80.3	52	45.6	31.5	14-59
14	40	23	57.5	41.5	14-55	32	80.0	20	62.5	20.5	10-52.5
20	108	57	52.8	21.5	10-44.5	90	83.3	46	51.1	18.5	8-43
21	101	52	51.5	33	9-48	78	77.2	39	50.0	28	7-45
23	142	77	54.2	26.5	9-45	127	89.4	71	55.9	20	8-42
28	172	92	53.5	28.5	8.5-43	146	84.9	80	54.8	29	10-43
31	198	104	52.5	27.5	10-47	178	89.9	96	53.9	22.5	9-45
33	127	63	49.6	20	8-46	115	90.6	58	50.4	19	8-46
Total IDA2	1618	840	51.9	29	10-49	1337	82.6	694	51.9	25	9-46
Total All	4610	2395	52.0	28	11-49	3812	82.7	1966	51.6	25	10-47

DA: diethylcarbamazine and albendazole; IDA1: ivermectin one dose, diethylcarbamazine and albendazole; IDA2: ivermectin two dose with DA; IQR: interquartile range
