# Supplementary material for: The safety of combined triple drug therapy with ivermectin, diethylcarbamazine and albendazole in the neglected tropical diseases co-endemic setting of Fiji: A cluster randomised trial
Source: PLoS Negl Trop Dis. 2020 Mar 16;14(3):e0008106. doi: 10.1371/journal.pntd.0008106 (PMC7098623; doi:10.1371/journal.pntd.0008106)
Supplement: S5 Table — DA: diethylcarbamazine and albendazole; IDA1: ivermectin one dose, diethylcarbamazine and albendazole; IDA2: ivermectin two dose with DA; Pop.: population; Med.: median; IQR: interquartile range. (PDF) [file pntd.0008106.s008.pdf]

S5 Table. Demographics of non-participants in 2017

Village	Pop.	Non-participants		Demographics Declined						Demographics Away					
	Total	Total		Total		Male		Age		Total		Male		Age	
	N	n	%	N	%	n	%	Med.	IQR	N	%	n	%	Med.	IQR
DA															
2	18	0	0	0	0	0	0	-	-	0	0	0	0	-	-
3	204	37	18.1	26	12.7	17	65.4	38	31-53	11	5.4	8	72.7	22	17-64
4	107	23	21.5	14	13.1	7	50.0	56	39-74	9	8.4	4	44.4	42	31-57
8	102	24	23.5	12	11.8	6	50.0	70	52.5-76.5	12	11.8	7	58.3	29	22.5-51.5
16	98	12	12.2	5	5.1	2	40.0	52	21-61	7	7.1	3	42.9	55	19-59
18	253	38	15.0	8	3.1	7	87.5	44	40-49	30	11.8	14	46.7	26	5-34
26	297	31	10.4	14	4.7	11	78.6	31	18-38	17	5.7	13	76.5	25	15-39
29	298	129	43.3	87	29.2	41	47.1	17	10-42	42	14.1	23	54.8	26	6-43
30	75	8	10.7	0	0	0	0	0	-	8	10.7	3	37.5	44	30-53.5
32	118	12	10.2	2	1.7	2	100	45	37-53	10	8.5	5	50.0	56.5	52-73
34	46	9	19.6	0	0	0	0	0	-	9	19.6	6	66.7	24	4-30
Total DA	1616	323	20.0	168	10.4	93	55.4	37	13-50.5	155	9.6	86	55.5	28	17-52
IDA1															
6	79	12	15.2	9	11.4	5	55.6	65	55-71	3	3.8	0	0	26	19-54
7	66	19	28.8	3	4.5	2	66.7	69	23-74	16	24.2	8	50.0	38	20.5-53.5
10	240	36	15.0	12	5.0	9	75.0	38	13.5-61	24	10.0	11	45.8	35	20.5-59.5
13	73	14	19.2	9	12.3	3	33.3	70	50-76	5	6.8	3	60.0	63	46-70
15	151	25	16.6	12	7.9	6	50.0	61	46.5-67	13	8.6	3	23.1	49	41-54
17	86	8	9.3	1	1.2	1	100	64	-	7	8.1	2	28.6	42	20-60
19	99	15	15.2	4	4.0	4	100	66	42.5-82	11	11.1	8	72.7	35	21-44
22	139	23	16.7	3	2.2	3	100	47	39-48	20	14.5	14	70.0	28	21-56
24	45	5	11.1	2	4.4	2	100	34	27-41	3	6.7	1	33.3	38	2-38
25	92	17	18.5	10	10.9	4	40.0	43.5	38-58	7	7.6	3	42.9	37	20-59
27	146	19	13.0	3	2.1	3	100	31	27-80	16	11.0	9	56.3	37	23.5-50
35	160	1	0.6	1	0.6	0	0	17	-	0	0	0	0	-	-
Total IDA1	1376	194	14.1	69	5.0	42	60.9	50	37-70	125	9.1	62	49.6	38	21-54
IDA2															
1	107	19	17.8	3	2.8	2	66.7	27	16-40	16	15.0	8	50.0	30	23.5-48
5	224	44	19.6	29	12.9	16	55.2	53	32-70	15	6.7	7	46.7	51	24-61
9	60	12	20.0	7	11.7	4	57.1	58	45-70	5	8.3	2	40.0	57	32-61
11	197	56	28.4	39	19.8	23	59.0	51	22-70	17	8.6	8	47.1	36	25-57
12	142	28	19.7	13	9.2	10	76.9	57	29-81	15	10.6	8	53.3	54	23-60
14	40	8	20.0	3	7.5	2	66.7	46	44-93	5	12.5	1	20.0	58	54-60
20	108	18	16.7	2	1.9	2	100	54.5	43-66	16	14.8	9	56.3	44	20-53
21	101	23	22.8	6	5.9	4	66.7	39	33-42	17	16.8	9	52.9	51	27-61
23	142	15	10.6	1	0.7	0	0	60	-	14	9.9	6	42.9	41.5	27-50
28	172	26	15.1	2	1.2	2	100	18.5	2-35	24	14.0	10	41.7	25	5.5-58
31	198	20	10.1	4	2.0	3	75.0	15	1.5-30.5	16	8.1	5	31.3	44	35-61
33	127	12	9.4	3	2.4	2	66.7	21	0-39	9	7.1	3	33.3	23	5-49
Total IDA2	1618	281	17.4	112	6.9	70	62.5	45	27-66	169	10.4	76	45.0	41	23-58
Total All	4610	798	17.3	349	7.6	205	58.7	41	21-60	449	9.7	224	49.9	36	21-55

DA: diethylcarbamazine and albendazole; IDA1: ivermectin one dose, diethylcarbamazine and albendazole; IDA2: ivermectin two dose with DA; Pop.: population; Med.: median; IQR: interquartile range
