# Supplementary material for: The safety of combined triple drug therapy with ivermectin, diethylcarbamazine and albendazole in the neglected tropical diseases co-endemic setting of Fiji: A cluster randomised trial
Source: PLoS Negl Trop Dis. 2020 Mar 16;14(3):e0008106. doi: 10.1371/journal.pntd.0008106 (PMC7098623; doi:10.1371/journal.pntd.0008106)
Supplement: S6 Table — DA: diethylcarbamazine and albendazole; IDA1: ivermectin one dose, diethylcarbamazine and albendazole; IDA2: ivermectin two dose with DA; IQR: interquartile range; LF: lymphatic filariasis; CFA +: circulating filarial antigen positive; Mf +: microfilariae positive; STH: soil- transmitted helminths; AE: adverse event. a Denominator tested for CFA excludes n = 60 declined and n = 93 ineligible. b Denominator for Mf excludes n = 11 unreadable smears, n = 71 declined and n = 93 ineligible. c Denominator for AE excludes n = 200 not treated and n = 14 lost to follow-up. (PDF) [file pntd.0008106.s009.pdf]

S6 Table. Baseline parasitic infection prevalence and adverse events by village

	Parasitic infection Prevalence												Safety		
	LF CFA +			LF Mf +			Scabies			STH			AE		
**Village**	N[a]	n	%	N[b]	n	%	N	n	%	N	n	%	N[c]	n	%
DA															
2	18	7	38.9	18	0	0	18	2	11.1	10	1	10.0	18	5	27.8
3	161	37	23.0	161	5	3.1	167	2	1.2	35	1	2.9	159	32	20.1
4	83	40	48.2	83	16	19.3	84	7	8.3	23	0	0	82	10	12.2
8	74	17	23.0	74	3	4.1	78	5	6.4	10	0	0	73	7	9.6
16	83	12	14.5	82	1	1.2	86	5	5.8	22	0	0	80	1	1.3
18	207	41	19.8	207	19	9.2	215	62	28.8	23	5	21.7	202	37	18.3
26	256	11	4.3	256	1	0.4	266	34	12.8	19	5	26.3	252	60	23.8
29	166	4	2.4	166	1	0.6	169	44	26.0	35	10	28.6	162	31	19.1
30	58	9	15.5	58	0	0	67	4	6.0	35	1	2.9	57	5	8.8
32	98	6	6.1	98	0	0	106	9	8.5	30	1	3.3	97	14	14.4
34	35	2	5.7	35	1	2.9	37	2	5.4	26	11	42.3	34	1	2.9
Total DA	1239	186	15.0	1238	47	3.8	1293	176	13.6	268	35	13.1	1216	204	16.8
IDA1															
6	67	19	28.4	66	6	9.1	67	6	9.0	17	0	0	66	15	22.7
7	47	13	27.7	44	3	6.8	47	8	17.0	4	0	0	46	13	28.3
10	194	35	18.0	193	7	3.6	204	12	5.9	33	2	6.1	198	34	17.2
13	59	14	23.7	50	2	4.0	59	1	1.7	13	2	15.4	58	10	17.2
15	121	17	14.0	121	6	5.0	126	36	28.6	27	0	0	122	25	20.5
17	75	18	24.0	74	12	16.2	78	8	10.3	17	3	17.6	75	11	14.7
19	81	1	1.2	81	0	0	84	16	19.0	16	3	18.8	73	23	31.5
22	112	17	15.2	111	4	3.6	116	27	23.5	16	0	0	107	12	11.2
24	38	1	2.6	38	0	0	40	2	5.0	20	12	60.0	36	3	8.3
25	72	4	5.6	72	2	2.8	75	3	4.0	19	7	36.8	67	21	31.3
27	117	3	2.6	117	1	0.9	127	11	8.7	68	14	20.6	115	8	7.0
35	159	3	1.9	159	1	0.6	159	50	31.4	110	16	14.5	155	35	22.6
Total IDA1	1142	145	12.7	1126	44	3.9	1182	180	15.2	360	59	16.4	1118	212	19.0
IDA2															
1	87	18	20.7	87	2	2.3	88	8	9.1	0	-	-	87	10	11.5
5	174	41	23.6	173	11	6.4	180	17	9.4	27	1	3.7	177	16	9.0
9	48	17	35.4	48	7	14.6	48	10	20.8	30	4	13.3	48	22	45.8
11	136	54	39.7	132	18	13.6	141	11	7.8	20	1	5.0	136	29	21.3
12	104	23	22.1	104	7	6.7	114	4	3.5	26	1	3.8	108	14	13.0
14	31	6	19.4	31	0	0	32	1	3.1	8	0	0	31	4	12.9
20	86	3	3.5	86	0	0	90	14	15.6	16	1	6.3	85	7	8.2
21	73	8	11.0	73	1	1.4	78	18	23.1	19	14	73.7	68	10	14.7
23	119	10	8.4	119	3	2.5	127	31	24.4	34	12	35.3	115	20	17.4
28	139	1	0.7	139	0	0	146	8	5.5	29	4	13.8	135	23	17.0
31	170	1	0.6	170	0	0	178	23	12.9	66	36	54.5	169	14	8.3
33	111	3	2.7	111	0	0	115	12	10.4	23	3	13.0	105	18	17.1
Total IDA2	1278	185	14.5	1273	49	3.8	1337	157	11.7	298	77	25.8	1264	187	14.8
Total All	3659	516	14.1	3637	140	3.8	3812	513	13.5	926	171	18.5	3598	600	16.7

DA: diethylcarbamazine and albendazole; IDA1: ivermectin one dose, diethylcarbamazine and albendazole; IDA2: ivermectin two dose with DA; IQR: interquartile range; LF: lymphatic filariasis; CFA +: circulating filarial antigen positive; Mf +: microfilariae positive; STH: soil- transmitted helminths; AE: adverse event.

[a] Denominator tested for CFA excludes n=60 declined and n=93 ineligible.

[b] Denominator for Mf excludes n=11 unreadable smears, n=71 declined and n=93 ineligible.

[c] Denominator for AE excludes n=200 not treated and n=14 lost to follow-up
