# Supplementary material for: The safety of combined triple drug therapy with ivermectin, diethylcarbamazine and albendazole in the neglected tropical diseases co-endemic setting of Fiji: A cluster randomised trial
Source: PLoS Negl Trop Dis. 2020 Mar 16;14(3):e0008106. doi: 10.1371/journal.pntd.0008106 (PMC7098623; doi:10.1371/journal.pntd.0008106)
Supplement: S7 Table — AE: adverse event; DA: diethylcarbamazine and albendazole; IDA: ivermectin, diethylcarbamazine and albendazole; Mod: moderate severity; NEC: not elsewhere classified. (PDF) [file pntd.0008106.s010.pdf]

S7 Table. List of reported adverse event symptoms with associated severity in order of frequency in participants treated for filariasis and followed up

	DA N=1216				IDA N=2382				Total N=3598					
**AE symptom**	Mild	Mod	Severe	Total DA	Mild	Mod	Severe	Total IDA	Mild	Mod	Severe	Total	% Total	% with AE
Any AE	195	5	3	203	364	28	5	397	559	33	8	600	16.7	
Fatigue	104	2	0	106	196	3	2	201	300	5	2	307	8.5	51.2
Headache	29	3	0	32	69	9	1	79	98	12	1	111	3.1	18.5
Dizziness	21	0	1	22	59	3	0	62	80	3	1	84	2.3	14.0
Nausea	26	1	0	27	45	3	0	48	71	4	0	75	2.1	12.5
Arthralgia	17	3	0	20	42	8	0	50	59	11	0	70	1.9	11.7
Myalgia	15	2	0	17	42	3	2	47	57	5	2	64	1.8	10.7
Muscle weakness	23	0	0	23	36	3	0	39	59	3	0	62	1.7	10.3
Pruritus	8	0	0	8	27	1	0	28	35	1	0	36	1.0	6.0
Diarrhoea	7	0	0	7	20	2	2	24	27	2	2	31	0.9	5.2
Vomiting	7	0	0	7	16	4	0	20	23	4	0	27	0.8	4.5
Abdominal pain	3	0	0	3	15	0	0	15	18	0	0	18	0.5	3.0
Cough	5	0	0	5	11	1	0	12	16	1	0	17	0.5	2.8
Local pain NEC	5	0	0	5	6	5	0	11	11	5	0	16	0.4	2.7
Rash	3	0	1	4	11	0	0	11	14	0	1	15	0.4	2.5
Dyspnoea	3	1	1	5	5	2	0	7	8	3	1	12	0.3	2.0
Fever	6	0	0	6	5	0	0	5	11	0	0	11	0.3	1.8
Abscess	5	0	0	5	4	0	0	4	9	0	0	9	0.3	1.5
Pain scrotum	3	0	0	3	5	0	0	5	8	0	0	8	0.2	1.3
Local swelling NEC	1	0	0	1	6	1	0	7	7	1	0	8	0.2	1.3
Chills	1	0	0	1	2	0	1	3	3	0	1	4	0.1	0.7
Skin pain	0	0	0	0	3	0	0	3	3	0	0	3	0.1	0.5
Swelling groin	1	0	0	1	2	0	0	2	3	0	0	3	0.1	0.5
Pain armpit	1	0	0	1	1	0	0	1	2	0	0	2	0.1	0.3
Nasopharyngitis	2	0	0	2	0	0	0	0	2	0	0	2	0.1	0.3
Swelling scrotum	0	0	0	0	2	0	0	2	2	0	0	2	0.1	0.3
Abdominal distension	0	0	0	0	2	0	0	2	2	0	0	2	0.1	0.3
Haemoptysis	0	0	0	0	1	1	0	2	1	1	0	2	0.1	0.3
Swelling armpit	1	0	0	1	0	0	0	0	1	0	0	1	0	0.2
Pain groin	1	0	0	1	0	0	0	0	1	0	0	1	0	0.2
Conjunctivitis	0	0	0	0	0	1	0	1	0	1	0	1	0	0.2
Insomnia	0	0	0	0	1	0	0	1	1	0	0	1	0	0.2
Hypoaesthesia	0	0	0	0	1	0	0	1	1	0	0	1	0	0.2
Infected scabies	0	0	0	0	1	0	0	1	1	0	0	1	0	0.2
Limb injury	0	0	0	0	0	1	0	1	0	1	0	1	0	0.2
Delusion	0	0	0	0	0	1	0	1	0	1	0	1	0	0.2
Per vaginal bleed	0	0	0	0	1	0	0	1	1	0	0	1	0	0.2

AE: adverse event; DA: diethylcarbamazine and albendazole; IDA: ivermectin, diethylcarbamazine and albendazole; Mod: moderate severity; NEC: not elsewhere classified
