# Supplementary material for: The safety of combined triple drug therapy with ivermectin, diethylcarbamazine and albendazole in the neglected tropical diseases co-endemic setting of Fiji: A cluster randomised trial
Source: PLoS Negl Trop Dis. 2020 Mar 16;14(3):e0008106. doi: 10.1371/journal.pntd.0008106 (PMC7098623; doi:10.1371/journal.pntd.0008106)
Supplement: Attachment — Submitted filename: Response_PLOSNTD_Review_2020-01-09.pdf [file pntd.0008106.s012.pdf]

Reviewer's Responses to Questions with Author's Response

Methods

<u>**Reviewer #1**</u>

The objectives are clearly articulated and the population appropriate for these objectives. The objectives of the paper are to evaluate the safety of IDA in Fiji, providing a detailed account of AEs following MDA in Fiji and an analysis of factors associated with their occurrence including filariasis, scabies, and STH infections. However, this subset of data from Fiji as part of the international study fundamentally compromises the power of the analysis to achieve its objectives of establishing if IDA materially alters the safety profile of DA. While there are unique co-infection rates in Fiji, the approach of the authors to answer safety would have been more informative if the analysis was based on coinfections rather than national boundaries and included data from the entire study.

Individual country studies were not powered to determine the difference of safety between DA and IDA as outlined in line 332. The overall safety results from 5 country studies have already been published.[13] This study provides additional information on the experience in Fiji that has a high rate of scabies. While data for co-infections of scabies and STH from all sites would have increased the strength of the findings, these data were not collected from all sites and therefore are not presented in this paper.

There are several other aspects of the methods employed for the main study and this sub-group analysis that are limiting for the stated objectives:

1. there are two fundamental issues missing from the rationale:
A. There is no discussion of the pharmacological aspects of adding ivermectin to this regimen to understand impact on metabolic pathways, risk of drug:drug interactions or transporter interactions, and extrinsic factors such as food effects. A statement on the pharmacology of the three agents would be the minimum expected.

We have included comments on the pharmacology of the three agents in the Introduction as follows (lines 124-126, page 6 and lines 142-144, page 7 respectively):

“The precise mechanisms of action of these medications on the filarial worm are not fully understood, but efficacy studies have determined macrofilaricidal and microfilaricidal activity when used in either two drug combination.”

Combining the three active agents into the one MDA combination, known as IDA, “does not create any drug-drug interactions, and” is superior to DA in clearing microfilaremia “at least” as long as 36 months after a single round of treatment.[8-"10"]

B. An evaluation of the impact on these drugs on pathogen/host response to treatments to contextualise the safety data or approach taken is also not presented to justify the methods chosen.

These evaluations would inform the safety methods employed, including laboratory assessments if required, and the nature and duration of adverse events to be of particular interest.

We had already described the action of the drugs on filaria and the host response (lines 144-146, page 7, Ref 11-12). We have now added a sentence on the relationship between treatment for scabies/STH and adverse events to contextualise the safety assessments conducted in the study, as follows (lines 159-160, page 7):

"Successful treatment of scabies and STH is expected to induce mild and transient symptoms, primarily pruritus (scabies) and abdominal pain (STH).[16,18]"

2. 2 days of active and 5 days of passive adverse event follow up should have been justified in the paper, particularly passive reporting as a method to fully elucidate safety differences. It appears practicality and cost driven rather than the optimal approach to establishing relative risk. As a result, the adverse event rates appear unexpectedly low for systematically collected data, even over such a short period of time.

The time period for adverse event follow-up is directly related to the known half-life, pharmacology of the drugs and timing of adverse events in previously conducted intensive pharmacokinetic studies. These studies found that peak drug levels and associated AEs occur within the first 12 hours even in LF uninfected individuals.[9-10] AEs associated with killing of Mf occur within the first 24 to 48 hours, and effects of adult worm death appear later and up to 7 days.[9-11] We have expanded the rationale for the time period of adverse events as follows (lines 252-262, pages 10-11):

Safety was assessed in two periods, consistent with the timing of AEs observed in previous studies.[8-11] "Active follow-up of all participants occurred daily in the first two days following treatment, when more severe symptoms are expected with death of microfilariae.[11] For continuity, participants were seen by the same nurse on the day of taking tablets and day 1 and 2 afterwards, and asked a standard open-ended question about their health. During days 3 to 7 after treatment," monitoring comprised of two activities: 1) participants were asked to notify a study representative if they were unwell and required assessment, and 2) participants previously identified as having an AE judged moderate or worse were assessed each day, with assessments only stopping if symptoms improved to mild severity or resolved completely (S2 Fig).

3. the rationale for not using a standardised toxicity grading scale to ensure consistency of grading across multiple sites, and the training provided for safety assessors to ensure standardisation of approach is also not described.

A standardised toxicity grading scale was used in this trial and this is referenced (Ref 27) following the description of the grading scale (lines 264-267, page 11). This scale has been used by multiple safety studies for LF (Ref 8 and 10). We have added a comment on training to increase clarity of standardisation amongst staff and across sites as follows (lines 250-252, page 10):

"A training package from the global study [13] was provided to all staff to ensure consistency of reporting between staff and across sites."

4. It should be stated if the analysis plan was signed off prior to database lock and unaltered post-lock, and that statistical consideration was made for this sub-group analysis.

We have included a comment as follows (lines 314-319, page 13):
"Data were analysed as prospectively planned and in accordance with International Conference on Harmonisation Statistical Principles for Clinical Trials Guideline E9. A statistical analysis plan was written for the main study and followed for the Fiji study. The Fiji site differs from the global study analysis by including children aged 2-4 years who received LF treatment with DA according to WHO guidelines."

5. The rationale for not blinding the study has also not been made and is a significant compromise to this design.

We have already listed unblinding of participants and assessors to treatment as a limitation of our study in the Discussion with a rationale for why blinding was not possible (lines 560-564, pages 26-27). We disagree with the reviewer that unblinding has significantly compromised our design of this community-based cluster randomised study.

6. Contraceptive measures for women of child bearing potential should be described.

We have provided further information as follows (lines 213-216, page 9):
"All women of child-bearing age were asked on the timing of their last menses. If menses was more than 4 weeks or unknown, a urine pregnancy test was offered. No contraceptive measures were provided or recommended, consistent with WHO policy for LF MDA."

7. It is assumed that a standard question was asked to elicit adverse events from participants. This should be stated.

The assumption is correct. We have included additional details in our response and additions to point #2 above (lines 250-262, pages 10-11).

8. The training provided to study nurses to ensure consistency between reviewers and grading of events should also be described. Did they all attend formalised training at an investigator meeting? Was this applied internationally?

We have addressed these points with additions made in response to point #3 above.

9. Were concurrent medications recorded?

Concurrent medications were only recorded for participants that experienced a serious adverse event.

10. Was regulatory approval received?

We have detailed regulatory approvals already in lines 336-342, page 14.

11. How were the variable requirements for fed and fasting state handled in the protocol?

In this community-based study there was no requirement to administer medication in relation to timing of food consumption. The goal was to replicate a realistic experience of LF mass drug administration by country programs. Timing medication around food consumption would be a barrier to adherence with directly observed therapy by local drug distributors.

12. The statistical testing performed should comply with ICHE9. A statement about compliance with Declaration of Helsinki would also be expected.

This has been addressed in response to point #4 above (lines 314-316, page 13).

Reviewer #2

Specific Points:
1) Line 145: this would be a good section to describe the age inclusion/exclusion differences from the parent study.

We have included this additional detail as follows (lines 202-205, page 9):

"Our study design differed from the global study by including all community members regardless of eligibility for LF treatment in order to allow community assessments for all three infections at both timepoints."

2) Lines 363-4: need the numbers of pruritus in <5 year old group give the comment about increased pruritis in the IDA group if disproportionately in the children <5 would be worth noting given they may have scabies not optimally treated with topicals and without ivermectin.

We have included a comment on the numbers of individuals with scabies in the IDA group who received permethrin instead of ivermectin, along with numbers of participants in DA group who didn't receive treatment until after AE monitoring period, as follows (lines 412-415, page 19):

"Thirty-two of the 305 (10.5%) participants with scabies in the IDA group were ineligible for ivermectin and received permethrin instead on day 0. The 164 participants with scabies in the DA group did not receive permethrin treatment until day 8, after safety monitoring period was completed."

We have also detailed the number of participants that received permethrin and experienced pruritus in the IDA group, as follows (lines 477-478, page 22):

"Only one participant who reported pruritus in the IDA group received permethrin."

Reviewer #3

The authors should clarify and delineate between the safety and efficacy on the main objective of the study. More insight and clarity is needed in understanding the study methods, the specific procedures involved in both intervention and data collection, how responses are transcribed and translated, how issues of language barriers had been addressed.
Authors have not pointed out specific hypothesis being tested.

The additional text added in response to Reviewer #1 provides further clarity regarding study methods and procedures. In regards to language and translation, we have added the following sentence to the Methods (lines 244-245, page 10):

"Written material was provided in English and the Fijian language, iTaukei, and staff members provided verbal translation in iTaukei and Rotuman as required."

Results

<u>**Reviewer #1**</u>

The rationale for the microfilariae levels chosen to report against should be provided. Mf presence or absence is the analysis of interest for adverse events incidence and severity but the number of infected people is small which limits the conclusions that can be drawn from the study.
There are too many subdivisions and subgroups in the demography. Suggest this is simplified and standardised to 0-28 day, 28 day to 2 year, 3 to 11 years, 12 to 17, 18 to 65, >65 for age and few subdivisions for Mf unless justified by the biology of response. Height and weight should have been collected and should be reported.

We believe that our age breakdown is meaningful, and corresponds to treatment groups and infection risk for LF, and therefore we do not wish to change our subgroups.
In regards to weight and height, we have added the following sentence to the Results (lines 354-355, page 15):

"The median height and weight for the 2161 participants aged 18 years and older was 169 cm (IQR 163-175 cm) and 84.7 kg (IQR 72.5-97.7 cm) respectively."

In line 318, it appears that 100 people who were not eligible for ivermectin received it. Under what circumstances and what happened to them?

The reviewer has mis-read the relevant sentence. We believe that the sentence is clear as written (lines 410-412, page 19):

"One person in the DA group received IDA and 123 in the IDA group received DA (due to exclusion from ivermectin because of weight and/or age)."

Caution should be expressed from the relative risk analysis in Table 3. There are too few patients and the risk of Type 1 or 2 errors is not accounted for in the authors presentation.

We have included the following text in our Discussion (lines 564-566, page 27):

"A further limitation of the study is the relatively small number of participants in our sub-analyses, meaning that cautious interpretation is required for risk differences."

Line 216 described drug/pathogen interactions as important for adverse events but it is more accurate to describe this as pathogen/host interactions as a result of drug efficacy.

We have edited lines 286-287, page 12 as follows:

From a safety perspective, "it was important to be able to relate potential effects of co-infection on drug efficacy and AEs."

Line 357 p value, if post hoc, should be declared as post hoc.

We have edited the sentence on line 467, page 22, as follows:

"In a post-hoc analysis of " participants with microfilaremia, the geometric mean Mf density was significantly higher in those that experienced an AE after treatment (357 Mf/ml, 95% CI 223-569 versus 131 Mf/ml, 95% CI 95-181, ttest P=0.0004).

Outcomes of SAEs are not given.

We have included the following to line 496, page 23:

"All three had complete resolution of their symptoms."

Reviewer #2

The ITT reporting of the age 2-5 group as having received ivermectin needs to be supplemented with an analysis of the "as treated" population given the pre-specification to treat the only >5 y/o children with ivermectin. This may or may not have an impact on analyzing the pruritus finding in the two regimens.

Our goal was to report the frequency of AEs experienced by a community when DA is recommended compared to IDA. Consistent with this goal we have analysed all treated within their assigned randomisation group, regardless of whether they were ineligible for ivermectin. We have now added an analysis of total AEs excluding children aged less than 5 years, as follows (lines 441-442, page 20).

"We observed a similar frequency of AEs when we excluded children aged less than 5 years (DA 17.0% versus IDA 17.3%, *P*=0.90)."

Table 2: suggest either an Asterix on the IDA column for the age 2-4 year old group or remove the numbers from the column given they did not receive IDA as per protocol exclusion.

We have added a superscript '[b]' to the heading 'Total treated LF and followed-up' with corresponding footnote as follows:

"[b] Participants received treatment as randomised except 1 in a DA village received IDA, and 121 in IDA villages received DA (ineligible for ivermectin due to weight and/or age)."

Reviewer #3

The tables provides substantial information on the findings of the study. However the tables could be summarized further to present the most essential information, the demographic information can be captured in the text.
Footnote on key descriptions of the table should be provided.

Our preference is to keep the demographic information in a table. We believe our footnotes adequately explain the content of the tables for our readers.

The discussion should elaborate on key issues of safety identified . The reported AEs should be exhaustively discussed to allay doubt etc.

We have added additional results describing timing of AEs as follows (lines 456-458, pages 21-22):

"Eighteen (3.2%) had an AE that persisted beyond 48 hrs following treatment and 44 (7.3%) experienced their first AE after 48 hrs."

We believe we have already outlined in the Discussion the key specifics of reported adverse events and any associations. We have added additional detail of type of adverse events to lines 527-528, page 25, as follows:

"Fatigue, headache, dizziness, nausea and arthralgia were the five most common symptoms reported."

Conclusions

Reviewer #1

The conclusions are not supported. There is Insufficient data in this sub-analysis and fundamental questions about the main study that undermine the objectives.

We disagree. We believe that our data provide valuable context-specific information to guide decision making locally, and are relevant to many other LF programs, particularly those in Pacific Island countries. Additionally, as indicated by Reviewer #2, we provide adverse event data in the setting of co-infections with scabies and STH, that are often co-endemic with LF in many tropical and sub-tropical settings. We note that the views of Reviewer#2 (see next point) and Reviewer #3 are supportive of our view and are in direct opposition to the view of Reviewer#1.

Reviewer #2

The conclusion of the parent study that triple drug combination is comparable in safety to the two drug standard of care is supported by the size of the study and the results presented in the multinational publication cited (Weil et al PLoS 2019.) In Fiji, adverse events were frequent albeit largely mild with only 3 unrelated SAE's. The results reported from Fiji do not demonstrate an effect of scabies or STH co-endemic infections on adverse events and this is important information for the endemic population of Fiji that remains resistant to MDA of the 2 drug regimen.

<u>Reviewer #3</u>

More information and discussion is required on how the data can be of help to advance our understanding safety in this study. The limitations of the analysis needs to come out clearly.
What level of confidence can we have in the finding that the results or findings in DA group compared to IDA, is not due to some form bias. How wide the gap is the confidence intervals as to make a strong case for judging the similarity between the two groups.

We have included 95% CI where appropriate in the results to aid the reader in interpretation of the confidence of our findings. For example, a section from the results below (lines 376-389, page 18):

"The prevalence of lymphatic filariasis across the two treatment groups was comparable as measured by detection of CFA (14.1%, 95% CI 11.3-17.5%) and Mf (3.8 %, 95% CI 2.6-5.6%, Table 1, S6 Table). The geometric mean Mf density was also similar between groups (DA 239 Mf/ml, range 17-4643, 95% CI 149-383, versus IDA 183 Mf/ml, range 17-9168, 95% CI 128-260). The intracluster correlation coefficient for Mf was 0.205. Rotuma had a higher CFA prevalence (24.8% versus Gau 6.1%; risk difference (RD) 19.3%, 95% CI 14.1-24.5%) and Mf prevalence (6.9% versus 1.6%; RD 5.6%, 95% CI 2.9-8.2%), but a comparable infection intensity (geometric mean Mf density Rotuma 206 Mf/ml, range 17-9168, 95% CI 148-288, versus Gau 181 Mf/ml, range 17-4492, 95% CI 106-308). Mf prevalence was higher in males (6% versus females 1.6%; RD 4.4%, 95% CI 2.4-6.3%), and those aged 35-49 years had the highest Mf prevalence (7.8%, 95% CI 4.9-12.3%). As the CFA score increased, the proportion with a positive test for Mf also significantly increased; 4.8% of grade 1 CFA were positive for Mf compared to 55.6% of those with CFA scores of 3 "(RD 50.6% 95% CI 41.5-59.6%)."

In addition, we have included a comment in our Discussion (lines 564-566, page 27):

"A further limitation of the study is the relatively small number of participants in our sub-analyses, meaning that cautious interpretation is required for risk differences."

Editorial and Data Presentation Modifications?

<u>Reviewer #1:</u> ***The paper is well written.***

<u>Reviewer #2:</u> ***(No Response)***

<u>Reviewer #3:</u> ***Minor revision***

Summary and General Comments

<u>Reviewer #1</u>

It is too ambitious to turn this national analysis into a declaration of safe or not safe for the new regimen. The study was not double blinded, and yet it could have been, the follow up duration was inadequate and should have driven by the biology of drug/host and pathogen/host factors. Further, non-standard methods of collecting adverse events and grading them were employed.

We have addressed Reviewer #1's comments and concerns in the individual points above.

<u>**Reviewer #2**</u>

The authors report on the safety of the standard of care regimen of diethylcarbamazine (DEC) and albendazole (DA regimen) versus the standard of care plus two doses of ivermectin (IDA1 and IDA2 regimen) and baseline status of scabies or soil-transmitted helminth infections (STH) from Fiji that apparently was part of a multi-national trial of the two regimens for treatment of lymphatic filariasis (LF). Fiji is endemic for LF and notably has previously received 10 rounds pf MDA with DA.

Clarification is needed between the design of the multi-national study that included Fiji and this manuscript from Fiji alone, now submitted and under review. The funding source and grant are the same yet the age of inclusion of children is different (>5 in Weil et al, >2 in Hardy et al) and the total numbers differ (3,419 in Weil et al and 3612 in Hardy et al) that cannot be reconciled by the 179 children enrolled in Hardy et al between 2-5 years of age.

We have responded to Reviewer #2's comments regarding clarity of methods and inserted additional information (lines 202-205, page 9) as outlined above.

The numbers reported from Fiji in the global study manuscript and in this manuscript can be reconciled. The two numbers referenced by Reviewer #2 above are not totals of the same group. The 3419 in the Weil paper refers to the total number treated for LF and followed up. If we add the 179 children aged 2-4 years that were treated and followed up, it gives a total of 3598 (the denominator in Table 2 in our manuscript). When we add the 14 participants treated and lost to follow-up to this figure, we come to 3612 (the denominator for total number treated for LF in Table1 and quoted by Reviewer #2 above).

The reporting of the data could also be more informative with further explanation of the as treated population. While an ITT analysis is appropriate to report safety results to avoid any interpretations based on post-randomisation events, the fact that children <5 were not treated per protocol design with ivermectin indicates that the as treated population should also be reported given ivermectin assignment is determined a priori by age.

As we have highlighted in our response to Reviewer #2 in the Results section above, we aimed to report the safety of a community intervention that would reflect as close as possible the experience of LF programs using IDA, which would include children aged 2-4 receiving DA. As mentioned above, we have added a comparison for total AEs excluding children aged less than 5 years (lines 441-442, page 20).

My suggestion is to present the analysis as originally described in the SAP but to add the "as treated data" for the <5 particularly in the sub-group with scabies since the next older age group had the largest burden of scabies and the numbers could be meaningful. If in

the end the original conclusions are un-changed, this would strengthen the conclusions of safety of the three-drug regimen in Fiji.

The numbers are too small to derive any difference to the conclusion of the safety of the three-drug regimen in Fiji. However, to provide clarity for the reader we have added the additional details for treatment and AEs in children less than 5 years (lines 434-439, page 20):

"There were 121 children aged less than 5 years in the IDA group that received DA and permethrin and were followed-up. Of these, six (5.0%) experienced an AE. There were 32 that had scabies and two (6.3%) experienced an AE. In contrast, there were 56 children aged less than 5 years in the DA group that received DA and were followed-up. Six (10.7%) experienced an AE. None of the 12 participants with scabies in this sub-group reported an AE, noting that they also did not receive scabies directed treatment until day 8."

Reviewer #3

The study is novel in the identification of the key issue of safety of combined therapy. The authors though needs to clearly exhaust this in the discussion to satisfy reader quest for information Give that cases of AEs have been reported, those cases all and all are critical to the outcome of the study. They must be exhaustively discussed and all issues clarified. What are the AEs caused by and what can we rely on to strengthen our belief in this.

In our response to Reviewer #1 in Methods section above, we have drawn attention to the expected cause of AEs when a participant is infected with LF (lines 144-146, page 7), and have added comments now for scabies and STH (lines 159-160, page 7). We have also expanded on the rationale for AE monitoring (lines 253-256, page 11). In addition, we have included further detail on the timing of AEs (lines 456-458, pages 21-22) and the type of AEs in the Discussion in response to Reviewer #3 comments in the Results section above (lines 527-528, page 25).

General Comments

The main title of the study had been stated in the secondary objectives and not the primary objective. This have not been clarified in context of using the given title. No hypothesis have been stated for testing. The authors need to delineate the issue of safety and efficacy. Their definitions in the context of the study objective as well as the procedures involved in their assessment needs further clarity and defined for more comprehension.

We have edited the title and abstract to improve clarity of the study objectives and findings. The aim of the study and focus of the manuscript is summarised at the end of the Introduction (lines 160-163, page 7). In regards to AE assessments, we have added further clarity in response to Reviewer #1 (lines 250-262, pages 10-11).

The methods section of the article needs to be reviewed for the flow of the information presented. In as much as the study aims to measure safety, the test of significance could have revealed critical and substantive information on the level of confidence we can have

when interpreting the results. Information on this is provided in a table but did come out clearly in the discussions

We have made edits to the Methods section in response to Reviewers' comments and believe the flow of information presented is now clear. We have also added additional 95% confidence intervals to the Results section to aid interpretation of the results.

What were the age limits of the eligible participants, and what specific tools were used to assess participants, was there a written questionnaire, which language was used as the medium of communication, was there an interpreter etc.

In regards to age limits of eligible participants, we have included the following additional detail (lines 202-205, page 9) in response to Reviewer #2:

"Our study design differed from the global study by including all community members regardless of eligibility for LF treatment in order to allow community assessments for all three infections at both timepoints."

In regards to language and translation, we have added the following sentence to the Methods (lines 244-245, page 10) in response to Reviewer #3:

"Written material was provided in English and the Fijian language, iTaukei, and staff members provided verbal translation in iTaukei and Rotuman as required."

Other Comments

L1-L3: You may to review the title to make it a more striking by making note of the use of key words as for the main objective of the study. An example title has been provided below for your review.

We have edited the title and short title as follows:

"The safety of triple drug therapy with ivermectin, diethylcarbamazine and albendazole in the neglected tropical diseases co-endemic setting of Fiji: a cluster randomised trial."

"Short title: Safety of triple drug therapy for neglected tropical diseases"

L4: Omit the abbreviation IDA

We have removed IDA from the titles as suggested.

L31-L38: The opening statement points to a condition and treatment efficacy than the main subject of safety in the title. Please delineate the two.

L34-L35: Information of existing treatment methods and safety should be mentioned. Had existing treatment strategy been evaluated for safety, and what were the findings.

We have addressed the two comments above and updated the Abstract background as follows (lines 36-40, page 3).

Lymphatic filariasis has remained endemic in Fiji despite repeated mass drug administration using "the well-established and safe combination of diethylcarbamazine and albendazole (DA) since 2002. In certain settings the addition of ivermectin to this combination (IDA) remains a safe strategy and is more efficacious. However, the safety has yet to be described in scabies and soil-transmitted helminth endemic settings like Fiji."

L39-42: The phrase cluster randomized trial mentioned in the title does not seem to be in fact a methodology, if so, can it then be omitted from the title altogether.

It is a recommendation of the CONSORT extension statement for cluster randomised trials to include "cluster randomised trial" in the title of the manuscript. It is a type of study design and therefore is relevant information in the title. To provide clarity we have edited the methodology of the abstract as follows (lines 42-47, page 3).

"Villages of Rotuma and Gau islands were randomised to either DA or IDA. Residents received weight-based treatment unblinded with standard exclusions. Participants were actively found and asked by a nurse about their health daily for the first two days and then asked to seek review for the next five days if unwell. Anyone with severe symptoms were reviewed by a doctor and any serious adverse event was reported to the Medical Monitor and Data Safety Monitoring Board."

L43-L48: The findings should discuss elaborately the key point of safety as the main subject here.

The infection prevalence details we have removed from the abstract and replaced with a summary of type of AEs experienced, as follows (lines 49-74, pages 3-4):

Of 3612 enrolled and eligible participants, 1216 were randomised to DA and 2396 to IDA. Age and sex in both groups were representative of the population. Over 99% "(3598)" of participants completed 7 days follow-up. Adverse events were reported by 600 participants (16.7%), distributed equally between treatment groups, with most graded as mild (93.2%). There were three serious adverse events, all judged not attributable to treatment "by an independent medical monitor. Fatigue was the most common symptom reported by 8.5%, with headache, dizziness, nausea and arthralgia being the next four most common symptoms." Adverse events were more likely in participants with microfilaremia (43.2% versus 15.7%), but adverse event frequency was not related to the presence of scabies or soil-transmitted helminth infection.

L44-L45: may as well belong to the section on Methodology.

It is a recommendation of the CONSORT extension statement for cluster randomised trials to include numbers randomised and analysed in the results section of the abstract.

L49-L54: This section requires more clarity as it points to the main subject of safety and efficacy because cases of AEs have been reported. How was it determined to conclude that

AEs especially the three serious cases, were not related to the treatment? This information is critical to the main purpose of the study and serve as a contentious point is objective assessment of the finding. Therefore, it needs to be clear and easy for the reader to understand.

We have addressed the concerns raised here in the re-wording of the Principal Findings section above (lines 49-74, pages 3-4).

L55-L58: Concluding statement should be made on reported cases of adverse events.

We have amended the abstract conclusion as follows (lines 76-80, page 4):

IDA has comparable safety to DA "with the same frequency of adverse events experienced following community mass drug administration." The presence of co-endemic infections did not increase adverse events. IDA can be used in community programs where preventative chemotherapy is needed for control of lymphatic filariasis and other neglected tropical diseases.

What is the current LF burden in Fiji?

We do not have country level LF prevalence data for Fiji, since there is substantial geographical heterogeneity in the burden we feel that this information would be misleading. We have added the last published Mf prevalence data for the Eastern Division in Fiji (lines 174-175, page 8), and removed the previously mentioned prevalence for the study sites since this is unpublished Fiji Ministry of Health and Medical Services data.

L98: list few names of countries, their regions that have achieved elimination targets, the and review the statement.

We have amended lines 117-118, page 6, and lines 128-131, page 6, as follows:

"The mosquito vector transmits the immature worms, known as microfilariae (Mf), from human to human in 61 endemic countries".

"Eleven countries, including Egypt, Thailand and Tonga, have achieved elimination targets and are under surveillance, but others, including eight countries in the Western Pacific Region such as Samoa, Philippines and Fiji, have not done so despite multiple rounds of MDA.[1]"

L106: please state briefly what is meant by adequate DA coverage?

We have amended lines 140-141 page 7 as follows:

However, despite reportedly adequate DA "treatment" coverage "above 65%", lymphatic filariasis has remained endemic in specific areas of Fiji.[1]

L134-L138: compare text with the following …. "The Eastern Division was chosen for the study because, it has the highest prevalence of filarial antigenemia in the country. Two of the divisions islands called Rotuma and Gau, were selected for the study":. …

We have amended the text as follows (lines 174-176, page 8):

"The Eastern Division was chosen for the study because it has the highest Mf prevalence in the country of 2.2% in 2007.[24] Two of the divisions islands, Rotuma and Gau, were selected study sites."

L144-L151: The flow of the information in this paragraph need to be reconstructed for more clarity and understanding.

We have amended the paragraph on trial design as follows (lines 181-205, pages 8-9).

"All 35 villages on the two islands agreed to participate in this study prior to randomisation. An independent statistician generated randomised treatment allocation using Stata software in a 1:1:1 ratio stratified by island to either DA, IDA1 (ivermectin administered with diethylcarbamazine and albendazole) or IDA2 (same as IDA1, plus a second dose of ivermectin on day 8). The IDA2 group was included to evaluate the community effectiveness of one versus two doses of ivermectin not reported here. Our study design differed from the global study by including all community members regardless of eligibility for LF treatment in order to allow community assessments for all three infections at both timepoints."

L189-L191: This needs more clarity… how could participant determine their own health, what had been done to minimize bias is response to those questions.

We have added further clarity in this section in response to Reviewer #1 in Methods above by adding comments on training and rationale and methods for adverse event monitoring (lines 250-262, pages 10 and 11) as follows:

The primary outcome of safety of IDA was evaluated at the individual and cluster levels. "A training package from the global study [13] was provided to all staff to ensure consistency of reporting between staff and across sites." Safety was assessed in two periods, consistent with the timing of AEs observed in previous studies.[8-"11] Active follow-up of all participants occurred daily in the first two days following treatment, when more severe symptoms are expected with death of microfilariae.[11] For continuity, participants were seen by the same nurse on the day of taking tablets and day 1 and 2 afterwards, and asked a standard open-ended question about their health. During days 3 to 7 after treatment," monitoring comprised of two activities: 1) participants were asked to notify a study representative if they were unwell and required assessment, and 2) participants previously identified as having an AE judged moderate or worse were assessed each day, with assessments only stopping if symptoms improved to mild severity or resolved completely (S2 Fig).

Additional Changes since Submission

Following submission of the manuscript in August, further data cleaning of the STH component has resulted in a reduction in total samples by 2, with a remaining sample size of 926. We have edited the text and tables (Tables 1-3, S6 Table) to reflect this change accordingly.

We have also updated the S1 Protocol (lines 754-756) and S3 Table (CONSORT) to represent the current version.

We have uploaded a striking still image to accompany our manuscript. We have a generic consent form signed from the parent, child, and staff member to use the photo for research purposes. If generic consent is not adequate, we have a still image of microfilariae that we could upload as an alternative.

Additional Changes on request of Editorial Staff following re-submission 8th January 2020

Please remove Competing Interests from the manuscript and ensure that they are added correctly to the Competing Interests section of the submission form.

Please remove financial details from the manuscript and ensure that they are added correctly to the Financial Disclosure section of the submission form

The described 'disclosure of interest statement' section as follows, has been removed from the manuscript

"**Disclosure of interest statement**
This study was supported in part by grant OPPGH5342 from the Bill & Melinda Gates Foundation to Washington University. The study was also supported in part by the Coalition for Operational Research on Neglected Tropical Diseases, which is funded at the Task Force for Global Health primarily by the Bill & Melinda Gates Foundation, by the United Kingdom Department for International Development, and by the United States Agency for International Development through its Neglected Tropical Diseases Program. Albendazole (produced and donated by GlaxoSmithKline) and diethylcarbamazine (produced and donated by Eisai Co., Ltd.) were obtained from Ministry of Health stocks in Fiji. Ivermectin was purchased from Merck Sharp Dohme (Australia) Pty. Ltd. and permethrin cream 5% was purchased from Pharmatec Wholesale Company Ltd. Fiji and manufactured by Glenmark Pharmaceuticals Ltd.
The funders and drug donors had no role in study design, data collection and analysis, decision to publish, or preparation of the manuscript."

Please remove Author Contributions from the manuscript and ensure that they are added correctly to the Author Contributions section under "Edit Author Details" in the "Add/Edit/Remove Author" section of the submission form

We have removed the following author contributions section (as follows) from the manuscript.

"**Author Contributions**
Conceptualization: Josaia Samuela, Andrew C. Steer, Gary J. Weil
Data curation: Myra Hardy
Formal analysis: Myra Hardy, Andrew C. Steer, Anneke C. Grobler
Funding acquisition: Gary J. Weil, Andrew C. Steer

Investigation: Myra Hardy
Methodology: Myra Hardy, Andrew C. Steer, John M. Kaldor, Gary J. Weil, Christopher L. King
Project administration: Myra Hardy, Josaia Samuela, Mike Kama, Meciusela Tuicakau, Lucia Romani, Andrew C. Steer
Resources: Josaia Samuela, Mike Kama, Gary J. Weil, Christopher L. King
Software: Myra Hardy
Supervision: Josaia Samuela, Andrew C. Steer, John M Kaldor, Margot J. Whitfeld, Leanne J. Robinson
Validation: Anneke C. Grobler
Visualization: Myra Hardy
Writing – original draft: Myra Hardy
Writing – review and editing: Myra Hardy, Josaia Samuela, Mike Kama, Meciusela Tuicakau, Lucia Romani, Margot J. Whitfeld, Christopher L. King, Gary J. Weil, Anneke C. Grobler, Leanne J. Robinson, John M. Kaldor, Andrew C. Steer"

We noticed that you used "unpublished" 1 time in the manuscript on page 31 (Reference 26). Please note that PLOS does not publish references to "unpublished" or "data not shown". More details can be found in our Data Policy here: Our full policy is located here: http://journals.plos.org/plosntds/s/data-availability. We ask that you please review this policy and provide the relevant data, either within the manuscript, the SI files, or in a public repository. Once those changes have been made, please add a paragraph at the end of your response to reviewers outlining the changes made due to this request. If you have any questions or if you are unsure how best to proceed, please don't hesitate to contact our staff for assistance

We have removed the following information due to source data being unpublished and updated references for manuscript:

"The most recent survey, in 2013, found the prevalence of filarial antigenemia to be 10.5% in Rotuma and 1.6% in Gau.[26]"
