# Supplementary material for: The safety of combined triple drug therapy with ivermectin, diethylcarbamazine and albendazole in the neglected tropical diseases co-endemic setting of Fiji: A cluster randomised trial
Source: PLoS Negl Trop Dis. 2020 Mar 16;14(3):e0008106. doi: 10.1371/journal.pntd.0008106 (PMC7098623; doi:10.1371/journal.pntd.0008106)
Supplement: Attachment — Submitted filename: The safety of combined ivermectin.docx [file pntd.0008106.s011.docx]

# The safety of combined triple therapy with ivermectin, diethylcarbamazine and albendazole in neglected tropical diseases co-endemic setting in Fiji: a cluster randomised trial.

**General Comments**

The main title of the study had been stated in the secondary objectives and not the primary objective. This have not been clarified in context of using the given title. No hypothesis have been stated for testing. The authors need to delineate the issue of safety and efficacy. Their definitions in the context of the study objective as well as the procedures involved in their assessment needs further clarity and defined for more comprehension.

The methods section of the article needs to be reviewed for the flow of the information presented. In as much as the study aims to measure safety, the test of significance could have revealed critical and substantive information on the level of confidence we can have when interpreting the results. Information on this is provided in a table but did come out clearly in the discussions

What were the age limits of the eligible participants, and what specific tools were used to assess participants, was there a written questionnaire, which language was used as the medium of communication, was there an interpreter etc.

**Other Comments**

# L1-L3: You may to review the title to make it a more striking by making note of the use of key words as for the main objective of the study. An example title has been provided below for your review.

L4**:** Omit the abbreviation **IDA**

L31-L38: The opening statement points to a condition and treatment efficacy than the main subject of safety in the title. Please delineate the two.

L34-L35: Information of existing treatment methods and safety should be mentioned. Had existing treatment strategy been evaluated for safety, and what were the findings.

L39-42: The phrase cluster randomized trial mentioned in the title does not seem to be in fact a methodology, if so, can it then be omitted from the title altogether.

L43-L48: The findings should discuss elaborately the key point of safety as the main subject here.

L44-L45: may as well belong to the section on Methodology.

L49-L54: This section requires more clarity as it points to the main subject of safety and efficacy because cases of AEs have been reported. How was it determined to conclude that AEs especially the three serious cases, were not related to the treatment? This information is critical to the main purpose of the study and serve as a contentious point is objective assessment of the finding. Therefore, it needs to be clear and easy for the reader to understand.

L55-L58: Concluding statement should be made on reported cases of adverse events

what is the current LF burden in Fiji?

L98: list few names of countries, their regions that have achieved elimination targets, the and review the statement.

L106: please state briefly what is meant by adequate DA coverage?

L134-L138: compare text with the following …. *“The Eastern Division was chosen for the study because, it has the highest prevalence of filarial antigenemia in the country. Two of the divisions islands called Rotuma and Gau, were selected for the study”*:. …

L144-L151: The flow of the information in this paragraph need to be reconstructed for more clarity and understanding.

L189-L191: This needs more clarity… how could participant determine their own health, what had been done to minimize bias is response to those questions.
