# Supplementary material for: The safety of combined triple drug therapy with ivermectin, diethylcarbamazine and albendazole in the neglected tropical diseases co-endemic setting of Fiji: A cluster randomised trial
Source: PLoS Negl Trop Dis. 2020 Mar 16;14(3):e0008106. doi: 10.1371/journal.pntd.0008106 (PMC7098623; doi:10.1371/journal.pntd.0008106)
Supplement: Attachment — Submitted filename: The safety of combined Ivermetin.docx [file pntd.0008106.s013.docx]

**Reviewer # 3.**

**General Comments**

This version of the article is much better written and its objectives are very reflected in both the Methodology and the Results. The methods and results sections are in conformity with the CONSORT (Consolidated Reporting Of trials) statement's extension to cluster randomized trials guidelines thereby making the study transparent and reproducible.  The approach to analysis is also appropriate.

Best wishes,

**Specific Comments**

**Methods**

The methods of the study are in conformity with the results and the authors made efforts to address concerns from the first review.

**Results**

The results sections is in conformity with the CONSORT (Consolidated Reporting Of trials) and much clearer for a general grasp of the findings.

**Conclusion**

A reasonable conclusion have been made as have been supported by the data. The authors can improve on the discussion by giving examples of similar studies and contrast their finding with this whether in agreement or not. Good Luck
